# A Fluorescent “Turn-On” Clutch Probe for Plasma Cell-Free DNA Identification from Lung Cancer Patients

**DOI:** 10.3390/nano12081262

**Published:** 2022-04-08

**Authors:** Lin Zhu, Dongxu Zhao, Lixin Xu, Meng Sun, Yueyue Song, Mingrui Liu, Menglin Li, Jinfeng Zhang

**Affiliations:** 1 Key Laboratory of Molecular Medicine and Biotherapy, School of Life Sciences, Beijing Institute of Technology, Beijing 100081, China; zhulin107@126.com (L.Z.); zhaodx@bit.edu.cn (D.Z.); xulixin1993@126.com (L.X.); m17863525419@163.com (M.S.); 3120201412@bit.edu.cn (Y.S.); 2 School of Materials Science and Engineering, Beijing Institute of Technology, Beijing 100081, China; 3220205144@bit.edu.cn

**Keywords:** fluorescent turn-on probe, circulating tumor nucleic acids, cell-free DNA, early cancer diagnosis, detection of double-stranded DNA

## Abstract

Early diagnosis of cancer is of paramount significance for the therapeutic intervention of cancers. Although the detection of circulating cell-free DNA (cfDNA) has emerged as a promising, minimally invasive approach for early cancer diagnosis, there is an urgent need to develop a highly sensitive and rapid method to precisely identify plasma cfDNA from clinical samples. Herein, we report a robust fluorescent “turn-on” clutch probe based on non-emissive QDs-Ru complexes to rapidly recognize EGFR gene mutation in plasma cfDNA from lung cancer patients. In this system, the initially quenched emission of QDs is recovered while the red emission of Ru(II) complexes is switched on. This is because the Ru(II) complexes can specifically intercalate into the double-stranded DNA (dsDNA) to form Ru-dsDNA complexes and simultaneously liberate free QDs from the QDs-Ru complexes, which leads to the occurrence of an overlaid red fluorescence. In short, the fluorescent “turn-on” clutch probe offers a specific, rapid, and sensitive paradigm for the recognition of plasma cfDNA biomarkers from clinical samples, providing a convenient and low-cost approach for the early diagnosis of cancer and other gene-mutated diseases.

## 1. Introduction

As one of the leading causes of morbidity and mortality worldwide, cancer remains a significant threat to global healthcare [1]. Although many therapeutic modalities have been developed to combat cancer, there is no treatment regimen to tackle cancer thoroughly with satisfactory performance in the clinic [2,3,4,5]. Therefore, the early diagnosis of cancer before its malignant progression and further metastasis is quite significant for increasing the survival rate of cancer patients [6]. An important focus of research to achieve this goal has been made on identifying alterations in tumor-related gene expression in clinical samples [7,8,9]. In particular, the detection of circulating tumor nucleic acids, especially circulating cell-free DNA (cfDNA), has been explored as a new but promising minimally invasive approach for early cancer diagnosis. The cfDNA is an emerging cancer-specific biomarker due to the mutation [10], aberrant methylation [11,12,13], microsatellite instability [14], and other abnormalities of cfDNA in the plasma and/or serum of cancer patients [15].

A vast number of diagnostic tests have been exploited to analyze cancer-specific plasma cfDNA for early cancer detection and therapeutic outcome evaluation [16,17]. Among them, DNA sequencing is one of the most widely available methods for cfDNA identification in clinical utilization. However, intrinsic biological interference, massive sample capacity, and high expense are critical limitations that hinder the further widespread implementation of DNA sequencing methods such as polymerase chain reaction (PCR) [18] and the “next-generation” sequencing (NGS) [19,20,21]. Recently, fluorescent techniques based on the quenching-recovering (“turn-on”) mechanism offer competitive superiority over DNA sequencing for cfDNA detection because of their high sensitivity, easy accessibility, good repeatability, as well as low cost [22,23,24]. Although attractive in principle, serious interference of biological or environmental background signals would substantially reduce current fluorescence imaging probes [25]. Therefore, developing a highly sensitive and reliable fluorescent probe to precisely identify plasma cfDNA from clinical samples is challenging yet urgently needed.

**Figure 1:**
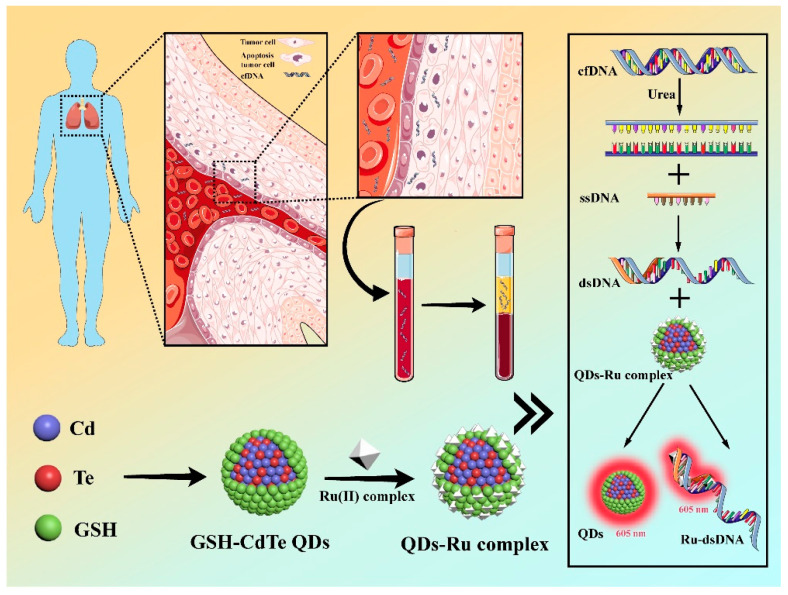
A schematic illustration of the QDs-Ru complexes consisted of water-dispersible GSH-CdTe QDs and fluorescence-quencher Ru(II) complexes as robust fluorescent “turn-on” clutch probes for the detection of cancer-specific cfDNA in patient plasma samples.

In this work, we developed a robust fluorescent “turn-on” clutch probe to rapidly detect lung cancer-specific cfDNA in patient plasma samples (Figure 1). Briefly, glutathione (GSH)-modified CdTe Quantum Dots (QDs) were interacting with a typical fluorescence-quencher Ru(II) complex [Ru(phen)_2_(dppz)]^2+^ to form a dual-component clutch probe (termed as QDs-Ru complexes), in which the emission of QDs could be entirely quenched by the Ru(II) complex via charge transfer. Fluorescent probes based on CdTe QDs have been widely applied as one of the most commonly used sensing candidates due to their unique tunable optical properties [26], simple synthesis and modification [27,28,29,30,31], and exceptionally high quantum yield [32]. Interestingly, the addition of the non-emissive QDs-Ru complexes into a double-stranded DNA (dsDNA) solution would render the fluorescence recovery of the QDs. The Ru(II) complex could bind dsDNA to form a Ru-dsDNA complex with a high affinity that would simultaneously liberate free QDs to turn on their red fluorescence. More importantly, the as-formed Ru-dsDNA complexes would also produce solid red fluorescence due to the prominently restrained solvent effects of the Ru(II) complex in aqueous solutions. By virtue of the overlaid red fluorescence from both QDs’ recovered emissions and the Ru(II) complexes’ emerged emissions, the as-prepared fluorescent “turn-on” clutch probe could be effectively applied to selectively recognize cancer-specific cfDNA with EGFR gene mutation from the blood plasma of lung cancer patients, showing great promise for rapid, sensitive, practicable identification of circulating tumor nucleic acids and further early cancer diagnosis.

## 2. Experimental Sections

### 2.1. Materials and Instruments

Tellurium (−200 mesh) and sodium borohydride (NaBH_4_) were purchased from Alfa Aesar. L-glutathione(GSH) and urea were purchased from Beijing BioDee Biotechnology Co. Ltd. (Beijing, China). Cadmium chloride hemipentahydrate (CdCl_2_·2.5H_2_O), orthoboric acid (H_3_BO_3_), disodium phosphate dodecahydrate (Na_2_HPO_4_·12H_2_O), sodium phosphate monobasic (NaH_2_PO_4_·2H_2_O), ethylenediaminetetraacetic acid (EDTA), hydrochloric acid (HCl), sodium hydroxide (NaOH), potassium chloride (KCl), sodium bicarbonate (NaHCO_3_), sodium chloride (NaCl), sodium citrate (C_6_H_5_Na_3_O_7_) and isopropanol were purchased from Beijing Chemical Industry Group Co., Ltd. (Beijing, China). All of the above were analytical reagent grade or better.

Plasmid Cas9 was kindly provided by the Yongjun Feng Group (Beijing Institute of Technology, Beijing, China), and bovine serum albumin (BSA) was purchased from Sigma-Aldrich (Shanghai, China). Different sequences of the single-stranded DNA (ssDNA) were synthesized from Taihe Biotechnology Co., Ltd. (Bejing, China). The lines of the oligonucleotides used in this work were shown as follows:

ssDNA: 5′-ATC AAG GAA TTA AGA GAA GCA ACA TCT CCG AAA-3′;

Complementary ssDNA (0 BM-ssDNA): 3′-TAG TTC CTT AAT TCT CTT CGT TGT AGA GGC TTT-5′

5-bases-mutated ssDNA (5 BM-ssDNA): 3′-TAG TTG GTT AAT TCA CGT CGT TCT AGA GGC TTT-5′

15-bases-deleted ssDNA (15 BD-ssDNA): 3′-TAG TTC TGT AGA GGC TTT-5′

Complementary Cas9 plasmid short ssDNA (0 BM-S-ssDNA): 5’-AAA TAG TCT ACG ATA AAA TGA AAG TCT AGA GGA TTC TCA-3′

Complementary Cas9 plasmid 1-base-mutated short ssDNA (1 BM-S-ssDNA): 5’-AAA TAG TCT AGG ATA AAA TGA AAG TCT AGA GGA TTC TCA-3′

Complementary Cas9 plasmid 3-bases-mutated short ssDNA (3 BM-S-ssDNA): 5’-ATA TAG TCT ACG ATT AAA TGA AAC TCT AGA GGA TTC TCA-3′

Complementary Cas9 plasmid 5-bases-mutated short ssDNA (5 BM-S-ssDNA): 5’-AAA TAG TGT ACG ATA ATA TGA ATC TCT ACA GGA TTC TCA-3′

Complementary EGFR exon-19-mutated ssDNA: 5’-TAG TTC CTT AAT TCT CTT CGT TGT AGA GGC TTT-3′

Complementary EGFR exon-20-mutated ssDNA: 5’-CCT GAG ACC TAG GGT CTT CCA CTC TTT CAA TTT TAA GGG-3′

The fluorescence emission spectrum was measured with a spectrophotometer (HORIBA Jobin Yvon, Paris, France). The fluorescence intensity was measured by exciting the sample at 390 nm. The UV-vis absorption spectrum was performed on a U-3900 spectrophotometer (Hitachi, Tokyo, Japan). Transmission electron microscopy (TEM) was obtained on a JEOL JEM-2100 electron microscope (Tokyo, Japan). The diameter of the synthesized compounds was measured by a Nano-ZS90 Laser Particle Size Analyzer (Malvern, UK). The pH value was measured by an AL104-IC PH meter (Mettler Toledo, Greifensee, Switzerland).

### 2.2. Synthesis of the GSH-Modified CdTe QDs (GSH-CdTe QDs)

To synthesize water-dispersible GSH-CdTe QDs, firstly, tellurium (Te) powder (63.8 mg) was reacted with NaBH_4_ (100 mg) in an N_2_ saturated deionized water (5 mL), producing a colorless solution of sodium hydrogen telluride (NaHTe). Then, CdCl_2_·2.5H_2_O (213.2 mg), GSH (368.5 mg), and 0.04% mercaptopropionic acid solution (50 mL) were mixed in a three-port flask, followed by the dropwise addition of NaOH to adjust the pH to 8.4–8.6 under magnetic stirring in a N_2_ atmosphere for 30 min. Afterward, the above-obtained NaHTe solution was rapidly injected into the Cd source precursor solution and slowly heated to 100 °C under N_2_ for another 5.5 h. At every 30 min interval, 100 μL samples were collected to detect the emission wavelength, and the reaction was stopped when the ideal emission wavelength was detected. The resulting GSH-CdTe QDs solution was then centrifuged at 8000 rpm/min for 15 min and subsequently washed with GSH and isopropanol three times. Finally, the GSH-CdTe QDs were dried into powder for further use.

The fluorescence quantum yield of the as-synthesized GSH-CdTe QDs samples was determined according to the following formula:

$$\phi_{x}=\phi_{s}\left(\frac{M_{x}}{M_{s}}\right){\left(\frac{\eta_{x}}{\eta_{s}}\right)}^{2}$$

*x*: GSH-CdTe QDs;
*s*: Rhodamine 6G;
$\phi_{x}$: The quantum yield of GSH-CdTe QDs;
$\phi_{s}$: The quantum yield of the reference substance rhodamine 6G;
$M_{x}$ and $M_{s}$: The ratios of the respective integrated area of fluorescence to the maximum absorbance at the excitation wavelength of GSH-CdTe QDs and the reference substance rhodamine 6G;
$\eta_{x}$ and $\eta_{s}$: The refractive index of GSH-CdTe QDs and reference substance rhodamine 6G.

### 2.3. Preparation of the QDs-Ru and Ru-dsDNA Complexes

When the molar ratio of GSH-CdTe QDs to Ru(II) complexes was 1:100, the fluorescence signal of the GSH-CdTe QDs would be almost wholly quenched. Therefore, the molar ratio was used to prepare the QDs-Ru complexes. In a typical procedure, 5 μL of the negatively charged GSH-CdTe QDs (1 μM) were mixed with 5 μL of the positively charged Ru(II) complex [Ru(phen)_2_(dppz)]^2+^ (1 μM) in PBS buffer solution (pH 7.4), where phen stands for 1,10-phenanthroline and dppz stands for dipyridophenazine at room temperature for 10 min, leading to the formation of the QDs-Ru complexes via electrostatic adsorption, whose fluorescence was detected at an excitation wavelength of 390 nm.

To form the Ru-dsDNA complexes, firstly, the specifically synthesized ssDNA (1 μg/mL) with the random sequence was added to the above-prepared QDs-Ru complexes at room temperature for 10 min incubation. Then, three different degrees of complementary sequences of the above-synthesized ssDNA with different concentrations were added into the mixture solution of ssDNA and the QDs-Ru complexes, which were incubated for another 10 min. Thus, this led to the quick self-assembly of dsDNA as well as simultaneous separation of GSH-CdTe QDs and Ru(II) complex from the QDs-Ru complexes. Meanwhile, the Ru-dsDNA complex would be subsequently formed due to the high affinity of the Ru(II) complex to dsDNA.

### 2.4. Denaturation of the dsDNA

Two kinds of DNA denaturants (urea, EDTA) with different concentrations were respectively mixed with Cas9 plasmid (6932 bp long dsDNA, L-dsDNA) and 33 bp short dsDNA (S-dsDNA) for 10 min incubation. Afterward, the QDs-Ru complexes were added into the denatured dsDNA for another 10 min incubation, followed by fluorescence detection.

In the case of the Cas9 plasmid (S-dsDNA), the denatured Cas9 plasmid and the addition 6 mol/L of urea were mixed with QDs-Ru complexes for 10 min incubation. Then, four different degrees of complementary short ssDNA (S-ssDNA) sequences of the Cas9 plasmid were added into the above mixture solution for another 10 min incubation, followed by fluorescence detection.

### 2.5. Biomolecular Interference Detection

An equal amount of dsDNA was added to the QDs-Ru complexes for 10 min incubation at room temperature. Subsequently, some biomolecules or ions such as Cl^−^, HPO_4_^2−^, citric acid, Na^+^, K^+^, Mg^2+^, Ca^2+^, and BSA were added to the above system. After 10 min, the fluorescence of the as-prepared samples was measured accordingly.

### 2.6. Detection of Clinical Samples

The urine samples from lung cancer patients were added to the QDs-Ru complexes solution. After incubation for 10 min, the fluorescence of the above-mixed solution was examined. Human blood samples were provided by Beijing Cancer Hospital from the provider with the age range of 20–60 years. The human blood samples were collected and centrifuged at 2000 rpm/min for 10 min. Then, the supernatant was transferred to a new centrifuge tube with another centrifugation at 12,000 rpm/min for 5 min to obtain plasma, which was stored at −20 °C for the following experiments. Then, 190 mL urea dissolved in PBS (9 mol/L) were added into 5 μL of the above plasma to denature the dsDNA contained in the plasma for stirring for 10 min. Afterward, the as-prepared QDs-Ru complexes were added. 10 min later, two kinds of complementary ssDNA (5 μL) with exon 19 and exon 20 mutation sites in the EGFR gene were added to the above-mixed solution, followed by fluorescence detection.

## 3. Results and Discussion

### 3.1. Preparation and Characterization of the Water-Dispersible GSH-CdTe QDs

**Figure 2:**
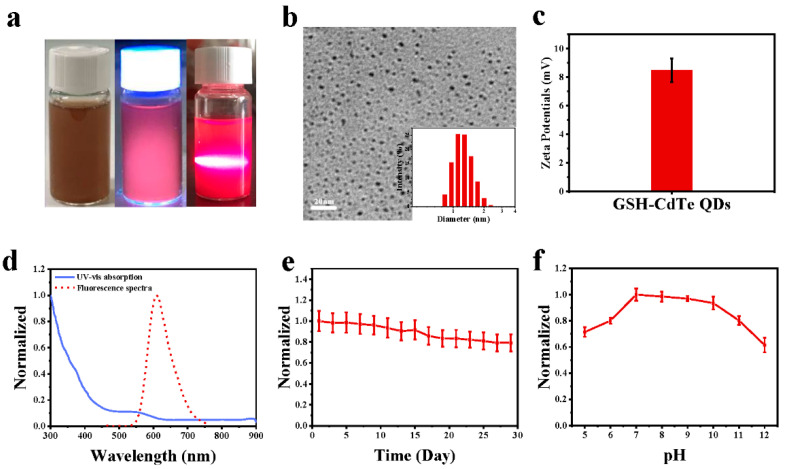
The characterization and stability of GSH-CdTe QDs. (**a**) Photographs of material color, red fluorescence, and Tyndall effect of QDs under natural light, UV, and IR light. (**b**) The TEM image and particle size of GSH-CdTe QDs. (**c**) The zeta potentials of GSH-CdTe QDs. (**d**) The UV-vis absorption and fluorescence spectra of GSH-CdTe QDs. (**e**) The stability of GSH-CdTe QDs via fluorescence spectra under 29 days. (**f**) The pH stability of GSH-CdTe QDs from 5 to 12.

The water-dispersible GSH-CdTe QDs were synthesized according to the previously reported work [33,34]. Typically, GSH was modified on the surface of the QDs by the formation of covalent bonds between the thiol of the GSH and the Cd atom at the CdTe QD surface, which would further improve the water dispersibility and bioavailability of the GSH modified QDs [26]. The as-synthesized GSH-CdTe QDs can be well dispersed in deionized water, and the typical Tyndall effect confirmed the existence of such QDs (Figure 2a). Figure 2b shows a transmission electron microscopy (TEM) image of the GSH-CdTe QDs, revealing their well-defined nanospheres with an average diameter of 2.63 ± 1.0 nm. The inset in Figure 2b presents a hydrodynamic diameter of 1.49 nm and a polydispersity index (PDI) value of 0.247 by dynamic light scattering measurement (DLS), which is almost consistent with the TEM result. Moreover, the as-obtained QDs exhibit a positive charge with a zeta potential of 8.49 mV (Figure 2c) could stabilize the QDs by electrostatic repulsion in an aqueous environment.

To investigate the optical properties of the GSH-CdTe QDs, the UV-vis absorption and fluorescence spectra of the QDs dispersed in deionized water were respectively measured and presented in Figure 2d. The GSH-CdTe QDs exhibit a strong absorption peak ranging from 300 nm to 450 nm while producing a maximum emission peak at 605nm with a robust red fluorescence tail extending to 750 nm [35,36]. A photograph of the GSH-CdTe QDs solution under UV irradiation also showed intense red emission (Figure 2a). The quantum yield of the water-dispersible GSH-CdTe QD was determined to be as high as 41.8% using rhodamine 6G (em: 550 nm, ex: 510 nm, QY = 95%) as the standard. Moreover, we also evaluated the photostability of the GSH-CdTe QDs over 29 days or in environments of different pH. As shown in Figure 2e,f, no apparent fluorescence quenching was found at the 605 nm emission peak during 29 days of storage, indicating the excellent chemical stability of the QDs. More interestingly, the fluorescence intensity of the GSH-CdTe QDs was stable over the pH range from 7 to 10 in PBS buffer solutions compared with that in deionized water.

In contrast, their fluorescence intensity was significantly decreased when the pH value was lower than 7 or higher than 10. The fluorescence quenching effect at a PH below 7 was ascribed to protonating the carboxyl groups on the surface of the GSH-CdTe QDs under acidic pHs [37], leading to the surface charge density change. In contrast, that at PH above 10 was attributed to removing part of the GSH surface ligand and under alkaline pHs [33], rendering QDs agglomeration, both of which lead to fluorescence quenching GSH-CdTe QDs.

### 3.2. Fluorescent “Turn-On” Properties of the Clutch Probe by Adding dsDNA

**Figure 3:**
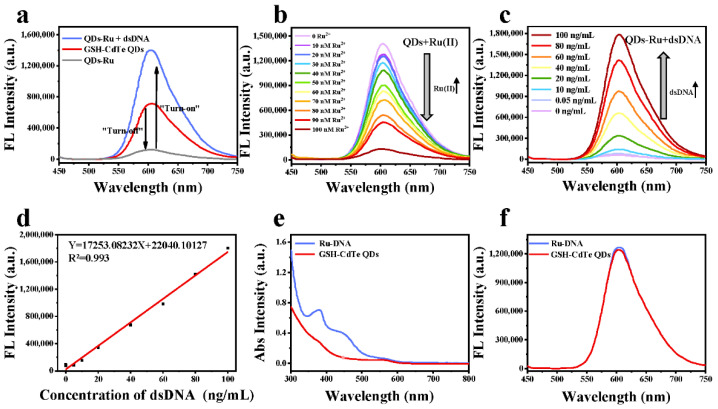
The fluorescent “turn-on” properties of the QDs-Ru complex. (**a**) The fluorescence quenching and recovery of QDs because of the Ru(II) complex and dsDNA interaction. (**b**) The fluorescence of GSH-CdTe QDs was quenched gradually with the increase of the Ru(II) complex’s concentration. (**c**) The fluorescence of GSH-CdTe QDs was recovered gradually with the increase of the concentration of dsDNA. (**d**) The linear relationship between the concentration of dsDNA and the recovered fluorescence intensity. The limit of detection is 0.06 ng/mL for dsDNA (*S*/*N* = 3). (**e**) The fluorescence spectrum and UV-vis absorption of GSH-CdTe QDs and Ru-dsDNA. (**f**) GSH-CdTe QDs and Ru-DNA complexes have similar emission wavelengths at the same excitation wavelength (ex: 390 nm, em: 605 nm).

Notably, the Ru(II) complex plays two significant roles in achieving the detection utilization of the as-designed clutch probe. On the one hand, the Ru(II) complexes can react with the GSH-CdTe QDs via electrostatic adsorption to form QDs-Ru complexes, resulting in fluorescence quenching of the QDs through a photo-induced electron transfer process [38,39], which was demonstrated in Figure 3a (data from the red line to the gray line). Notably, free Ru(II) complexes suffer from high susceptibility to water and the resultant fluorescence quenching effect, whereas Ru(II) complexes inserted into the dsDNA show strong red emission (data from the gray line to the blue line shown in Figure 3a) because the hydrophobic environment within the double-helix structures of dsDNA could prevent the Ru(II) complexes from being totally exposed to water and considerably avoid the solvent effect in aqueous solutions [40]. Therefore, the Ru(II) complexes can serve as an excellent clutch for dsDNA to form Ru-dsDNA complexes and concurrently release free GSH-CdTe QDs from the QDs-Ru complexes.

Impressively, as the concentration of added Ru(II) complexes increased, the fluorescence intensity of the GSH-CdTe QDs gradually decreased (Figure 3b). When the molar ratio of GSH-CdTe QDs to Ru(II) complexes was 1:100, the fluorescence signal of the GSH-CdTe QDs would be substantially quenched. Thus, a molar ratio was used to prepare the QDs-Ru complexes. On the other hand, the Ru(II) complex, regarded as a typical fluorescence switch for DNA molecules, can specifically intercalate into the major or minor grooves of the double-stranded DNA (dsDNA) to form Ru-dsDNA complexes, leading to the emergence of Ru(II) complexes’ fluorescence. As presented in Figure 3c,d, the recovered fluorescence of a mixture of QDs-Ru complexes and dsDNA gradually enhanced along with the increase of dsDNA concentration, where a well proportional linear relationship between the concentration of the added dsDNA and the recovered fluorescence intensity of the probe system could be found [41,42]. Furthermore, the detection limit of such a probe system was determined to be 0.06 ng/mL for dsDNA under the 3× signal-noise ratio (*S*/*N* = 3).

The absorption spectra of Ru-DNA and QDs were respectively depicted in Figure 3e, where several peaks in the visible region of Ru-DNA are attributed to the characteristic absorption bands from Ru complexes [43]. Most attractively, the same range of the absorption wavelength at 300–500 nm and almost the same emission peak at 605 nm of the Ru-dsDNA complexes and the GSH-CdTe QDs were indicated in Figure 3e,f. The overlaid “turn-on” red fluorescence stemming from the recovered emission of free GSH-CdTe QDs and emerged emission of the Ru(II) complexes could be simultaneously excited by a single wavelength at 390 nm, significantly improving the sensitivity and practicability of the as-designed probe in the following clinical samples identification.

After verifying that dsDNA can efficiently recover the fluorescence of the GSH-CdTe QDs, we next evaluated the influence of dsDNA concentration or sequence lengths on the fluorescence recovery capabilities. Since the cfDNA in human blood is intrinsically dsDNA fragments with different sequence lengths ranging from 100 bp to more than 2 million bp [44], both a short dsDNA (33 bp) and a long plasmid dsDNA (6932 bp) were respectively used to “turn-on” the fluorescence of the QDs-Ru complexes. It can be seen from Table 1 that the fluorescence intensity of the short dsDNA (33 bp) is comparable to that of the long dsDNA (6932 bp) at the same concentration. This result indicates that the response capacity of the fluorescent “turn-on” clutch probe is highly dependent on the concentration of the to-be-detected dsDNA instead of its sequence length. This is because the interacting driving force between Ru(II) complexes and dsDNA only relies on the number of grooves within DNA double-helix structures, consisting of four different base pairs [45,46]. In other words, the fluorescence intensity of the to-be-detected dsDNA only depends on the concentration of dsDNA rather than the sequence lengths or base compositions of dsDNA.

### 3.3. Fluorescent “Turn-On” Properties of the Clutch Probe by Successively Introducing ssDNA and Its Corresponding Complementary ssDNA

**Figure 4:**
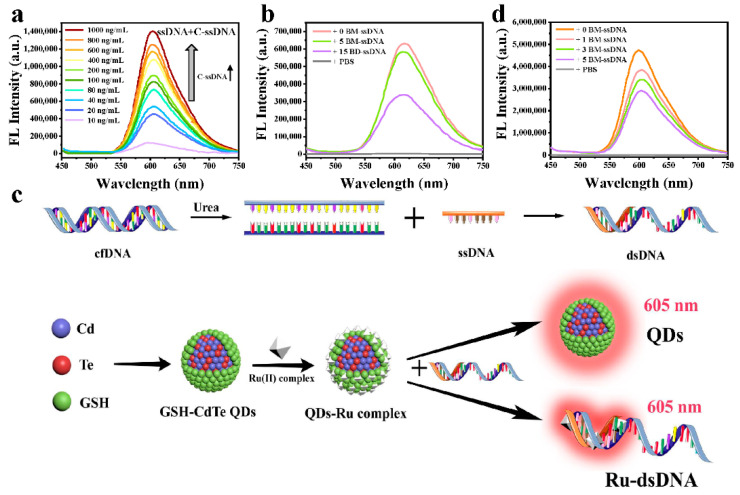
The specificity and mechanisms of the clutch probe. (**a**) The fluorescence of QDs is recovered with the increase of complementary ssDNA’s (C-ssDNA) concentrations. (**b**) The fluorescence spectra of QDs and Ru-DNA complexes after interaction with different complementarity of two ssDNA (0BM-ssDNA: fully complementary ssDNA; 5BM-ssDNA: 5 bases mismatched ssDNA; 15BD-ssDNA: 15 bases deleted ssDNA). (**c**) Mechanism of specific recognition by fluorescent probes. (**d**) The fluorescence spectra of QDs and Ru-DNA complexes after interaction with different complementarity of Cas9 plasmid L-dsDNA and specific Cas9 plasmid L-ssDNA (0BM-ssDNA: fully complementary ssDNA; 1BM-ssDNA: 1 base mismatched ssDNA; 3BM-ssDNA: 3 bases mismatched ssDNA; 5BM-ssDNA: 5 bases mismatched ssDNA).

Apart from detecting the dsDNA, we next investigated if the quenched fluorescence of QDs-Ru complexes could be “turned on” by successfully adding single-stranded DNA (ssDNA) and its corresponding complementary ssDNA. As shown in Figure 4a, upon beforehand adding ssDNA to the QDs-Ru complexes solution, the fluorescence intensity of the clutch probe gradually enhanced with the increase of the complementary ssDNA’s (C-ssDNA) concentration. As the amount of the added C-ssDNA increases, a growing number of dsDNA will be assembled, which further precisely clutches the Ru(II) complexes from the non-emissive QDs-Ru complexes to form the fluorescent Ru-dsDNA complexes and simultaneously liberate the fluorescent GSH-CdTe QDs.

It is well documented that the cfDNA in the blood of cancer patients generally has DNA bases mutation (BM-DNA) and DNA bases deletion (BD-DNA), which could change the structure of dsDNA [47,48]. Therefore, we will further verify whether the fluorescent “turn-on” clutch probe can detect BM-DNA and BD-DNA. First, we individually synthesized a ssDNA with a sequence of 5′-ATC AAG GAA TTA AGA GAA GCA ACA TCT CCG AAA-3′ and its corresponding complementary ssDNA (0 BM-ssDNA) with a sequence of 3′-TAG TTC CTT AAT TCT CTT CGT TGT AGA GGC TTT-5′, complementary 5-bases-mutated ssDNA (5 BM-ssDNA) with a sequence of 3′-TAG TTG GTT AAT TCA CGT CGT TCT AGA GGC TTT-5′, and complementary 15-bases-deleted ssDNA (15 BD-ssDNA) with a sequence of 3′-TAG TTC TGT AGA GGC TTT-5′. Then, after adding the as-synthesized ssDNA into the QDs-Ru complexes, the above three types of the complementary ssDNA were respectively added into the mixture solution. As expected, it can be found from Figure 4b that the higher the complementary degree between the ssDNA and its complementary ssDNA, the more vigorous the fluorescence intensity of the probe system could be detected. That means the clutch probe’s excellent fluorescent “turn-on” capability became functional by adding ssDNA and its corresponding complementary ssDNA.

In addition, for the practical identification of the cancer-specific cfDNA, the initial double helix structure of the long dsDNA (L-dsDNA, such as the cfDNA) should be unwound into long ssDNA (L-ssDNA) via DNA denaturant. Subsequently, L-ssDNA was specifically re-assembled with a deliberately designed short ssDNA (S-ssDNA) into a hybrid DNA possessing a short segment of dsDNA (S-dsDNA). In such a process, the “off”-stated clutch probe could be recovered into “on”-stated in the presence of the short dsDNA segment—this mechanism is illustrated in Figure 4c. In this case, urea, as a common DNA denaturant, was chosen to break the double helix structure of the L-dsDNA. It is also worth noting that the concentration of the denaturant urea added in the L-dsDNA-containing solution is critical. Because at that given concentration, the L-dsDNA can be unwound into L-ssDNA while the follow-up re-assembled S-dsDNA will not be denatured.

After monitoring the fluorescence changes of the QDs-Ru complexes individually incubated with an L-dsDNA or an S-dsDNA at different urea concentrations, we selected 9 mol/L of urea as the denaturation concentration toward L-dsDNA. At that concentration, the fluorescence of the L-dsDNA group rapidly decreased while the fluorescence of the S-dsDNA group was still robust (Figure S1).

After confirming the denaturation concentration, we used 9 mol/L urea to break the double-helix structure of a Cas9 plasmid L-dsDNA and then added the QDs-Ru complexes. Subsequently, the above mixture solution added four deliberately designed complementary Cas9 plasmid S-ssDNA with different sequences towards a specific Cas9 plasmid L-ssDNA segment. The sequences of the four complementary Cas9 plasmids S-ssDNA are shown as follows: 0 BM-S-ssDNA with the sequence of 5′-AAA TAG TCT ACG ATA AAA TGA AAG TCT AGA GGA TTC TCA-3′; 1 BM-S-ssDNA with the sequence of 5′-AAA TAG TCT AGG ATA AAA TGA AAG TCT AGA GGA TTC TCA-3′; 3 BM-S-ssDNA with the sequence of 5′-ATA TAG TCT ACG ATT AAA TGA AAC TCT AGA GGA TTC TCA-3′ and 5 BM-S-ssDNA with the sequence of 5′-AAA TAG TGT ACG ATA ATA TGA ATC TCT ACA GGA TTC TCA-3′.

As presented in Figure 4d, on the one hand, when compared to the group that added only PBS, the other four groups which introduced complementary Cas9 plasmid S-ssDNA with different sequences show noticeable fluorescence enhancements, suggesting the successful formation of the S-dsDNA in the opened L-ssDNA. Furthermore, the fluorescence gradually enhanced with the increase of the complementary degree, which is in line with the results in Figure 4b. These results demonstrated the excellent potential of the fluorescent “turn-on” clutch probe for cfDNA detection.

### 3.4. Detection of Clinic Plasma cfDNA from Lung Cancer Patients

**Figure 5:**
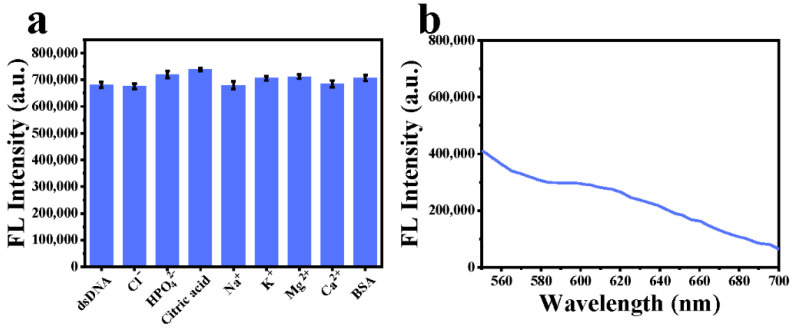
The detection of clinical samples. (**a**) Biomolecular interference detection with Cl^−^, HPO_4_^2−^, citric acid, Na^+^, K^+^, Mg^2+^, Ca^2+^ and BSA, respectively. (**b**) Fluorescence spectra of fluorescent probes after interaction with urine samples.

To investigate the feasibility of the fluorescent “turn-on” clutch probe for clinical application, we first evaluate the biomolecules interference effect in this system which is usually found in practical plasma detection [49,50]. As shown in Figure 5a, there is a negligible influence on the detected fluorescence when various biomolecules or ions, including Cl^−^, HPO_4_^2−^, citric acid, Na^+^, K^+^, Mg^2+^, Ca^2+^, and BSA were respectively added to the dsDNA and GSH-CdTe QDs mixture. On the other hand, urine and plasma are the two most commonly utilized clinical test samples due to their high association with some metabolic mechanisms of diseases [51,52]. Therefore, we first evaluated the fluorescent “turn-on” property at 605 nm in urine samples from lung cancer patients in this work. Interestingly, there is no noticeable fluorescence enhancement at 605 nm (Figure 5b), suggesting that either the urine samples contain no cfDNA or the cfDNA content within the urine is extremely low.

Alternatively, we then evaluated whether the as-prepared probe could be applied to detect the cancer-specific cfDNA in the plasma of cancer patients. It has been well-reported in the clinic that the epidermal growth factor receptor (EGFR) gene mutations occur most frequently in non-small cell lung cancer patients. Remarkably, approximately 60% of such cancer patients possess a mutation in exon 19 or exon 20 of the EGFR gene [53]. Based on these findings, we deliberately designed complementary EGFR exon-19-mutated or exon-20-mutated ssDNA to pair with the unwound EGFR exon-19-or exon-20-mutated cfDNA in plasma samples, which combined with the clutch probe to detect EGFR mutation in lung cancer patients. After successively adding the QDs-Ru complexes and the as-designed exon-mutated ssDNA, a higher fluorescence intensity recorded in the plasma samples represents a higher complementary degree to the mutant sequences in the EGFR gene. For practical application, we prepared 30 clinical plasma samples, 20 of which were collected from lung cancer patients (sample number is 1–20) and 10 of which were collected from the healthy person as negative controls. The detection results of EGFR exon 19 and exon 20 mutations from the clinical samples are summarized in Table 2 and Table 3. It is found that samples 2, 3, 4, 5, 8, 11, 13, 14, 18 in Table 2 and samples 4, 17, and 18 in Table 3 show a significant difference between the plasma fluorescence from the lung cancer patient and healthy person, showing that the cfDNA in the above samples, respectively, had a high complementary degree to the EGFR exon 19 and exon 20 mutations in lung cancer patients.

It is worth noting that we cannot negate the effect of the concentration of dsDNA in the blood samples on the fluorescence signal values. However, whether it is the better complementarity with the target dsDNA strand or the higher concentration of the detected dsDNA, it has a positive effect on the final detection results, which implies that more tumor-specific dsDNA exited and could be detected in cancer patients. Collectively, a total of 12 samples with EGFR mutations in cfDNA could be identified by the fluorescent “turn-on” DNA clutch probe, demonstrating the positivity rate of EGFR mutations in lung cancer patients is 60%, and the mutation probability of exon 19 is higher than that of exon 20 in EGFR gene, both of which are consistent with the clinical reports. More attractively, the cancer-specific mutant cfDNA could be distinguished from non-mutant type in lung cancer patients by only using as little as 5 μL unhandled plasma, where the plasma volume used here is significantly less than that required for a routine clinical plasma test (e.g., 0.2–2 mL), making the as-designed probe more practicable [54,55,56].

## 4. Conclusions

In summary, we established a new but reliable strategy for plasma cancer-specific cfDNA identification in lung cancer patients, which is based on a fluorescent “turn-on” DNA clutch probe (QDs-Ru complexes) via integration of red fluorescence from both QDs’ recovered and Ru(II) complexes’ emerged emissions. Particularly, the as-prepared fluorescent probe could be effectively applied to selectively recognize EGFR gene mutation in plasma cfDNA due to the high affinity of the Ru(II) complex towards double-stranded DNA. More importantly, only a minimal volume of plasma sample (e.g., 5 uL) was required to be used for a typical run of cfDNA detection by applying the as-designed strategy, which is much less than that of a routine clinical plasma test (e.g., 0.2–2 mL). In short, the fluorescent “turn-on” clutch probe offers a rapid, sensitive, practicable paradigm for recognizing plasma cfDNA biomarkers from clinical samples with easy accessibility and low cost, which also shows great promise for early diagnosis of cancer and other gene-mutated diseases.

## Figures and Tables

**Table 1:** The Fluorescence intensities of the QDs-Ru complexes were adding dsDNA fragments with different sequence lengths and different concentrations.

	0.5 ng/mL	5 ng/mL	10 ng/mL	20 ng/mL	40 ng/mL	60 ng/mL	80 ng/mL
Short dsDNA (33 bp)	151,900 ± 6883.5	157,790 ± 3680.9	173,570 ± 9251.5	209,880 ± 5014.1	273,980 ± 8090.8	323,070 ± 4491.8	424,740 ± 5339.4
Long plasmid dsDNA (6932 bp)	151,180 ± 7485.4	151,790 ± 5923.3	171,740 ± 5895.5	203,090 ± 7346.2	273,090 ± 5808.3	306,078 ± 3851.5	424,190 ± 3246.2

**Table 2:** The detection results of EGFR exon 19 mutations from the clinical samples.

Sample Number	Fluorescence Intensity of Plasma Samples from Lung Cancer Patients (Value CP)	Mean Fluorescence Intensity of 10 Plasma Samples from Heathy Person (Value HP)	(Value CP-Value HP)/Value HP (%)	Significance Test (*p*)
1	2,331,856.66	2,393,280.33	−2.56651	/
2	3,427,146.66	/	43.19871	***
3	2,971,850	/	24.17476	**
4	2,844,531	/	18.8549	**
5	2,932,493.33	/	22.53029	**
6	2,265,600	/	−5.33495	/
7	2,274,700	/	−4.95472	/
8	2,714,736.66	/	13.43162	*
9	2,108,196.66	/	−11.9118	/
10	2,309,500	/	−3.50065	/
11	3,049,036.66	/	27.3999	***
12	2,250,156.66	/	−5.98023	/
13	2,708,510	/	13.17145	*
14	3,096,290	/	29.37431	***
15	2,266,550	/	−5.29526	/
16	2,265,250	/	−5.34958	/
17	2,036,547	/	−14.9056	/
18	2,755,478.66	/	15.13397	*
19	2,318,096.66	/	−3.14145	/
20	2,325,440	/	−2.83462	/

* *p* < 0.05, ** *p* < 0.01, *** *p* < 0.001.

**Table 3:** The detection results of EGFR exon 20 mutations from the clinical samples.

Sample Number	Fluorescence Intensity of Plasma Samples from Lung Cancer Patients (Value CP)	Mean Fluorescence Intensity of 10 Plasma Samples from Heathy Person (Value HP)	(Value CP-Value HP)/Value HP (%)	Significance Test (*p*)
1	2,285,445.66	2,205,912.698	3.605445	/
2	2,255,669.33	/	2.255603	/
3	2,011,476	/	−8.81434	/
4	2,698,380	/	22.32488	***
5	2,232,551	/	1.207586	/
6	2,144,325	/	−2.79194	/
7	2,210,035	/	0.186875	/
8	2,019,885.33	/	−8.43312	/
9	1,977,845	/	−10.3389	/
10	2,144,789.66	/	−2.77087	/
11	2,254,776	/	2.215106	/
12	2,155,698	/	−2.27637	/
13	2,144,478.66	/	−2.78497	/
14	2,236,698.33	/	1.395596	/
15	2,166,458	/	−1.78859	/
16	2,215,936.33	/	0.454398	/
17	2,622,463.33	/	18.88337	***
18	2,780,916.66	/	26.06649	***
19	2,155,638	/	−2.27909	/
20	2,286,542	/	3.655145	/

*** *p* < 0.001.

## Data Availability

Not applicable.

## Supplementary Materials

The following supporting information can be downloaded at: https://www.mdpi.com/article/10.3390/nano12081262/s1, Figure S1: The fluorescence spectra of different concentrations of urea with L-dsDNA and S-dsDNA.

## References

[1] Cline B., Delahunty I., Xie J. (2019). Nanoparticles to mediate X-ray-induced photodynamic therapy and Cherenkov radiation photodynamic therapy. WIREs Nanomed. Nanobiotechnol..

[2] Fitzmaurice C., Abate D., Abbasi N., Abbastabar H., Abd-Allah F., Abdel-Rahman O., Abdelalim A., Abdoli A., Abdollahpour I., Abdulle A.S. (2019). Global, Regional, and National Cancer Incidence, Mortality, Years of Life Lost, Years Lived with Disability, and Disability-Adjusted Life-Years for 29 Cancer Groups, 1990 to 2017: A Systematic Analysis for the Global Burden of Disease Study. JAMA Oncol..

[3] Tu L., Liao Z., Luo Z., Wu Y.-L., Herrmann A., Huo S. (2021). Ultrasound-controlled drug release and drug activation for cancer therapy. Exploration.

[4] Fang F., Zhu L., Li M., Song Y., Sun M., Zhao D., Zhang J. (2021). Thermally Activated Delayed Fluorescence Material: An Emerging Class of Metal-Free Luminophores for Biomedical Applications. Adv. Sci..

[5] Fang F., Yuan Y., Wan Y., Li J., Song Y., Chen W.-C., Zhao D., Chi Y., Li M., Lee C.-S. (2022). Near-Infrared Thermally Activated Delayed Fluorescence Nanoparticle: A Metal-Free Photosensitizer for Two-Photon-Activated Photodynamic Therapy at the Cell and Small Animal Level. Small.

[6] Sullivan F.M., Mair F.S., Anderson W., Armory P., Briggs A., Chew C., Dorward A., Haughney J., Hogarth F., Kendrick D. (2020). Earlier diagnosis of lung cancer in a randomised trial of an autoantibody blood test followed by imaging. Eur. Respir. J..

[7] Lin D., Wu Q., Qiu S., Chen G., Feng S., Chen R., Zeng H. (2019). Label-free liquid biopsy based on blood circulating DNA detection using SERS-based nanotechnology for nasopharyngeal cancer screening. Nanomedicine.

[8] Wang Y., Dong L., Zhao J., Jalalah M., Al-Assiri M.S., Harraz F.A., Cao Y. (2021). Proximity-constructed bifunctional DNA probes for identification of stem-like biomarker in breast cancer. Sens. Actuators B Chem..

[9] Hamfjord J., Guren T.K., Dajani O., Johansen J.S., Glimelius B., Sorbye H., Pfeiffer P., Lingjærde O.C., Tveit K.M., Kure E.H. (2019). Total circulating cell-free DNA as a prognostic biomarker in metastatic colorectal cancer before first-line oxaliplatin-based chemotherapy. Ann. Oncol..

[10] Haseltine J., Offin M., Myers M.L., Makhnin A., Adamski A., Li H., Li M., Shaffer T., Henderson S., Shen R. (2019). Tumor volumetric correlation with plasma cell free DNA (cfDNA) mutation detection in metastatic lung cancers. J. Clin. Oncol..

[11] Luo H., Wei W., Ye Z., Zheng J., Xu R.-H. (2021). Liquid Biopsy of Methylation Biomarkers in Cell-Free DNA. Trends Mol. Med..

[12] Barault L., Amatu A., Siravegna G., Ponzetti A., Moran S., Cassingena A., Mussolin B., Falcomatà C., Binder A.M., Cristiano C. (2017). Discovery of methylated circulating DNA biomarkers for comprehensive non-invasive monitoring of treatment response in metastatic colorectal cancer. Gut.

[13] Warton K., Samimi G. (2015). Methylation of cell-free circulating DNA in the diagnosis of cancer. Front. Mol. Biosci..

[14] Han X., Zhang S., Zhou D.C., Wang D., He X., Yuan D., Li R., He J., Duan X., Wendl M.C. (2021). MSIsensor-ct: Microsatellite instability detection using cfDNA sequencing data. Brief. Bioinform..

[15] Gainetdinov I.V., Kapitskaya K.Y., Rykova E.Y., Ponomaryova A.A., Cherdyntseva N.V., Vlassov V.V., Laktionov P.P., Azhikina T.L. (2016). Hypomethylation of human-specific family of LINE-1 retrotransposons in circulating DNA of lung cancer patients. Lung Cancer.

[16] Howell J.A., Khan S.A., Knapp S., Thursz M.R., Sharma R. (2016). The clinical role of circulating free tumor DNA in gastrointestinal malignancy. Transl. Res..

[17] Caneira C.R.F., Soares R.R.G., Pinto I.F., Mueller-Landau H.S., Azevedo A.M., Chu V., Conde J.P. (2019). Development of a rapid bead-based microfluidic platform for DNA hybridization using single- and multi-mode interactions for probe immobilization. Sens. Actuators B Chem..

[18] Santos E.S., Talebi T.N., Raez L.E., Quintero C.A., Walker G., Farias M., Ramachandran K., Gordian E., Gomez J., Singal R. (2010). Free circulating DNA by RT-PCR as predictor for chemotherapy response in newly diagnosed patients (pts) with advanced non-small cell lung cancer (NSCLC). J. Clin. Oncol..

[19] Das J., Ivanov I., Sargent E.H., Kelley S.O. (2016). DNA Clutch Probes for Circulating Tumor DNA Analysis. J. Am. Chem. Soc..

[20] Heitzer E., Haque I.S., Roberts C.E.S., Speicher M.R. (2018). Current and future perspectives of liquid biopsies in genomics-driven oncology. Nat. Rev. Genet..

[21] Ahlquist D.A. (2018). Universal cancer screening: Revolutionary, rational, and realizable. NPJ Precis. Oncol..

[22] Ma K., Xie W., Liu W., Wang L., Wang D., Tang B.Z. (2021). Graphene Oxide Based Fluorescent DNA Aptasensor for Liver Cancer Diagnosis and Therapy. Adv. Funct. Mater..

[23] Du Y., Lai Y., Liu J.Y., Diao J. (2021). Epigenetic Quantification of DNA 5-Hydroxymethylcytosine Using DNA Hybridization-Based Single-Molecule Immunofluorescent Imaging. Small Methods.

[24] Li Z., Mao G., Du M., Tian S., Niu L., Ji X., He Z. (2019). A fluorometric turn-on aptasensor for mucin 1 based on signal amplification via a hybridization chain reaction and the interaction between a luminescent ruthenium(II) complex and CdZnTeS quantum dots. Mikrochim. Acta Mater..

[25] Miao Y., Lv J., Yan G. (2017). Hybrid detection of target sequence DNA based on phosphorescence resonance energy transfer. Biosens. Bioelectron..

[26] Korram J., Dewangan L., Karbhal I., Nagwanshi R., Vaishanav S.K., Ghosh K.K., Satnami M.L. (2020). CdTe QD-based inhibition and reactivation assay of acetylcholinesterase for the detection of organophosphorus pesticides. RSC Adv..

[27] Yong K.-T., Law W.-C., Roy I., Jing Z., Huang H., Swihart M.T., Prasad P.N. (2011). Aqueous phase synthesis of CdTe quantum dots for biophotonics. J. Biophotonics.

[28] Moulick A., Milosavljevic V., Vlachova J., Podgajny R., Hynek D., Kopel P., Adam V. (2017). Using CdTe/ZnSe core/shell quantum dots to detect DNA and damage to DNA. Int. J. Nanomed..

[29] Das P., Ganguly S., Margel S., Gedanken A. (2021). Tailor made magnetic nanolights: Fabrication to cancer theranostics applications. Nanoscale Adv..

[30] Wang F.-T., Wang L.-N., Xu J., Huang K.-J., Wu X. (2021). Synthesis and modification of carbon dots for advanced biosensing application. Analyst.

[31] Das P., Ganguly S., Margel S., Gedanken A. (2021). Immobilization of Heteroatom-Doped Carbon Dots onto Nonpolar Plastics for Antifogging, Antioxidant, and Food Monitoring Applications. Langmuir.

[32] Park J.C., Choi S.Y., Yang M.Y., Nan L., Na H., Lee H.N., Chung H.J., Hong C.A., Nam Y.S. (2019). Subnanomolar FRET-Based DNA Assay Using Thermally Stable Phosphorothioated DNA-Functionalized Quantum Dots. ACS Appl. Mater. Interfaces.

[33] Wang J., Li D., Liu X., Qiu Y., Peng X., Huang L., Wena H., Hu J. (2018). One-pot synthesis of highly luminescent N-acetyl-l-cysteine-capped CdTe quantum dots and their size effect on the detection of glutathione. New J. Chem..

[34] Liu Y.-F., Yu J.-S. (2009). Selective synthesis of CdTe and high luminescence CdTe/CdS quantum dots: The effect of ligands. J. Colloid Interface Sci..

[35] Zhang Z., Rogers C.R., Weiss E.A. (2020). Energy Transfer from CdS QDs to a Photogenerated Pd Complex Enhances the Rate and Selectivity of a Pd-Photocatalyzed Heck Reaction. J. Am. Chem. Soc..

[36] Medintz I.L., Pons T., Trammell S.A., Grimes A.F., English D.S., Blanco-Canosa J.B., Dawson P.E., Mattoussi H. (2008). Interactions between Redox Complexes and Semiconductor Quantum Dots Coupled via a Peptide Bridge. J. Am. Chem. Soc..

[37] Yang Q., Li Q., Li H., Li F. (2021). pH-Response Quantum Dots with Orange-Red Emission for Monitoring the Residue, Distribution, and Variation of an Organophosphorus Pesticide in an Agricultural Crop. J. Agric. Food Chem..

[38] Jia F., Wang S., Man Y., Kumar P., Liu B. (2019). Recent Developments in the Interactions of Classic Intercalated Ruthenium Compounds: [Ru(bpy)₂dppz]^2+^ and [Ru(phen)₂dppz]^2+^ with a DNA Molecule. Molecules.

[39] Zhang C., Hu Q., Wu S., Chen F. (2020). Selective determination of DNA based on the fluorescence recovery of carbon dots quenched by Ru(bpy)_2_(dppz)^2+^. Talanta.

[40] Zhang Y., Hou D., Zhao B., Li C., Wang X., Xu L., Long T. (2021). Ratiometric Fluorescence Detection of DNA Based on the Inner Filter Effect of Ru(bpy)_2_(dppx)^2+^ toward Silicon Nanodots. ACS Omega.

[41] Ling L.-S., Song G.-W., He Z.-K., Liu H.-Z., Zeng Y.-E. (1999). A Novel Method to Determine DNA by Use of Molecular “Light Switch” of Ru(phen)_2_(dppz)^2+^. Microchem. J..

[42] Zhao D., Chan W.H., He Z., Qiu T. (2009). Quantum Dot−Ruthenium Complex Dyads: Recognition of Double-Strand DNA through Dual-Color Fluorescence Detection. Anal. Chem..

[43] Yang C., Zhou Q., Jiao Z., Zhao H., Huang C.-H., Zhu B.-Z., Su H. (2021). Ultrafast excited state dynamics and light-switching of [Ru(phen)_2_(dppz)]^2+^ in G-quadruplex DNA. Chem. Comm..

[44] Amintas S., Vendrely V., Dupin C., Buscail L., Laurent C., Bournet B., Merlio J.-P., Bedel A., Moreau-Gaudry F., Boutin J. (2021). Next-Generation Cancer Biomarkers: Extracellular Vesicle DNA as a Circulating Surrogate of Tumor DNA. Front. Cell Dev. Biol..

[45] Ni J., Yang W., Wang Q., Luo F., Guo L., Qiu B., Lin Z., Yang H. (2018). Homogeneous and label-free electrochemiluminescence aptasensor based on the difference of electrostatic interaction and exonuclease-assisted target recycling amplification. Biosens. Bioelectron..

[46] Huang X., Bian X., Chen L., Guo L., Qiu B., Lin Z. (2021). Highly Sensitive Homogeneous Electrochemiluminescence Biosensor for Alkaline Phosphatase Detection Based on Click Chemistry-Triggered Branched Hybridization Chain Reaction. Anal. Chem..

[47] Topkaya S.N., Serindere G., Ozder M. (2015). Determination of DNA Hypermethylation Using Anti-cancer Drug-Temozolomide. Electroanalysis.

[48] Yan X.-L., Xue X.-X., Deng X.-M., Jian Y.-T., Luo J., Jiang M.-M., Zheng X.-J. (2020). Chemiluminescence strategy induced by HRP-sandwich structure based on strand displacement for sensitive detection of DNA methyltransferase. Microchem. J..

[49] Iqbal H., Yang T., Li T., Zhang M., Ke H., Ding D., Deng Y., Chen H. (2020). Serum protein-based nanoparticles for cancer diagnosis and treatment. J. Control. Release.

[50] Bergant M., Ščančar J., Milačič R. (2020). Kinetics of interaction of Cr(VI) and Cr(III) with serum constituents and detection of Cr species in human serum at physiological concentration levels. Talanta.

[51] Goetsch H.E., Zhao L., Gnegy M., Imperiale M.J., Love N.G., Wigginton K.R. (2018). Fate of the Urinary Tract Virus BK Human Polyomavirus in Source-Separated Urine. Appl. Environ. Microbiol..

[52] Zhou L., Gan N., Wu Y., Hu F., Lin J., Cao Y., Wu D. (2018). Multiplex detection of quality indicator molecule targets in urine using programmable hairpin probes based on a simple double-T type microchip electrophoresis platform and isothermal polymerase-catalyzed target recycling. Analyst.

[53] Xue X., Asuquo I., Hong L., Gao J., Dong Z., Pang L., Jiang T., Meng M., Fan J., Wen J. (2020). Catalog of Lung Cancer Gene Mutations Among Chinese Patients. Front. Oncol..

[54] Liu Y., Wu H., Zhang W., Zhang H., Chen H., Zhou G., Gu Y. (2019). Endonuclease-assisted hydrogel bead array for digital analysis of circulating tumor DNA methylation. Sens. Actuators B Chem..

[55] Ohira T., Sakai K., Matsubayashi J., Kajiwara N., Kakihana M., Hagiwara M., Hibi M., Yoshida K., Maeda J., Ohtani K. (2016). Tumor volume determines the feasibility of cell-free DNA sequencing for mutation detection in non-small cell lung cancer. Cancer Sci..

[56] Pécuchet N., Zonta E., Didelot A., Combe P., Thibault C., Gibault L., Lours C., Rozenholc Y., Taly V., Laurent-Puig P. (2016). Base-Position Error Rate Analysis of Next-Generation Sequencing Applied to Circulating Tumor DNA in Non-Small Cell Lung Cancer: A Prospective Study. PLoS Med..

