# Supplementary material for: A Fluorescent “Turn-On” Clutch Probe for Plasma Cell-Free DNA Identification from Lung Cancer Patients
Source: Nanomaterials (Basel). 2022 Apr 8;12(8):1262. doi: 10.3390/nano12081262 (PMC9027387; doi:10.3390/nano12081262)
Supplement: Supplementary file 1 [file nanomaterials-12-01262-s001.zip › nanomaterials-1623348-SI.pdf]

Supporting Information

A Fluorescent "Turn-On" Clutch Probe for Plasma Cell-Free DNA Identification from Lung Cancer Patients

Lin Zhu [1,†], Dongxu Zhao [1,†], Lixin Xu [1], Meng Sun [1], Yueyue Song [1], Mingrui Liu [2], Menglin Li [2,*] and Jinfeng Zhang [1,*]

[1] Key Laboratory of Molecular Medicine and Biotherapy, School of Life Sciences, Beijing Institute of Technology, Beijing 100081, China; zhulin107@126.com (L.Z.); zhaodx@bit.edu.cn (D.Z.); xulixin1993@126.com (L.X.); m17863525419@163.com (M.S.); 3120201412@bit.edu.cn (Y.S.)

[2] School of Materials Science and Engineering, Beijing Institute of Technology, Beijing 100081, China; 3220205144@bit.edu.cn (M.L.)

* Correspondence: menglinli90@bit.edu.cn (M.L.); jfzhang@bit.edu.cn (J.Z.)

† These authors contributed equally to this work.

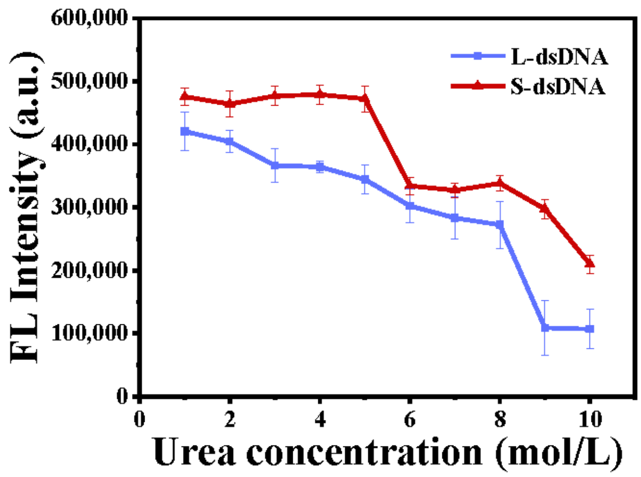

Figure S1. The fluorescence spectra of different concentrations of urea with L-dsDNA and S-dsDNA.
